# Structure of the Archaeal Pab87 Peptidase Reveals a Novel Self-Compartmentalizing Protease Family

**DOI:** 10.1371/journal.pone.0004712

**Published:** 2009-03-05

**Authors:** Vanessa Delfosse, Eric Girard, Catherine Birck, Michaël Delmarcelle, Marc Delarue, Olivier Poch, Patrick Schultz, Claudine Mayer

**Affiliations:** 1 Centre de Recherche des Cordeliers, LRMA, INSERM UMR-S 872, Université Pierre et Marie Curie, Paris, France; 2 Institut de Biologie Structurale UMR5075 CEA-CNRS-UJF, Grenoble, France; 3 Département de Biologie et Génomique Structurales, IGBMC, Illkirch, France; 4 Centre d'Ingénierie des Protéines, Université de Liège, Institut de Chimie B6, Sart-Tilman, Belgium; 5 Département de Biologie Structurale et Chimie, Institut Pasteur, CNRS URA 2185, Paris, France; University of Queensland, Australia

## Abstract

Self-compartmentalizing proteases orchestrate protein turnover through an original architecture characterized by a central catalytic chamber. Here we report the first structure of an archaeal member of a new self-compartmentalizing protease family forming a cubic-shaped octamer with *D*_4_ symmetry and referred to as CubicO. We solved the structure of the *Pyrococcus abyssi* Pab87 protein at 2.2 Å resolution using the anomalous signal of the high-phasing-power lanthanide derivative Lu-HPDO3A. A 20 Å wide channel runs through this supramolecular assembly of 0.4 MDa, giving access to a 60 Å wide central chamber holding the eight active sites. Surprisingly, activity assays revealed that Pab87 degrades specifically d-amino acid containing peptides, which have never been observed in archaea. Genomic context of the Pab87 gene showed that it is surrounded by genes involved in the amino acid/peptide transport or metabolism. We propose that CubicO proteases are involved in the processing of d-peptides from environmental origins.

## Introduction

**Figure 1:**
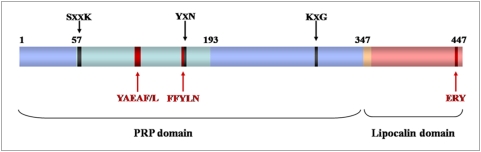
Primary structure of the CubicO proteases. Schematic representation of the CubicO protease sequences. The PRP (serine protease) α/β and all-helical regions are in light blue and cyan, respectively, the linking helix in orange and the lipocalin domain in salmon. Numbering refers to the Pab87 sequence. The three PRP conserved motifs are indicated in black, the three CubicO specific motifs in red.

Intracellular protein breakdown is a universal process that implicates protein degradation carried out by high-molecular-weight self-compartmentalizing proteases [1], [2]. They belong to different MEROPS families [3] but have converged towards the same barrel-shaped architecture. Compartmentalization confines the peptidase activity to inner cavities only accessible to unfolded polypeptides. Initial proteolysis is fulfilled by energy-dependent proteasome in eukaryotes and archaea [4] or by the equivalent bacterial counterparts, HslVU [5], ClpAP [6], or Lon [7], which generate peptides of about 10 amino acids long. In addition to the energy-dependent proteasome systems, energy-independent protease complexes have been described to further process and degrade the peptides produced by proteasomes. Organisms diverged in terms of peptide hydrolysis processes and have their own specific pool of different energy-independent proteases that functionally complements the proteasome activity [8]. Structural studies of such complexes, that putatively take part in the degradation of small oligopeptides, reveal several types of organization. The 720 kDa Tricorn protease from *Thermoplasma acidophilum* contains six subunits associated into rings with *D*_3_ symmetry and possesses trypsin- and chymotrypsin-like activities [9]. A larger assembly has been observed *in vivo* for Tricorn where 20 hexamers form a 14.6 MDa icosahedral capsid, whose physiological role is unknown [10]. The TET aminopeptidases are present in organisms where Tricorn is not found [11]. The crystal structures of archaeal *Pyrococcus horikoshii* PhTET1 and PhTET2 have revealed that these proteases adopt a tetrahedral shape containing twelve subunits [12], [13]. Like for Tricorn, TET can form a supramolecular assembly of 800 kDa composed of 24 subunits and referred to as octahedron, only observed *in vivo* yet [14]. TppII is the only energy-independent self-compartmentalizing protease found exclusively in eukaryotes and consists of a particle of more than 1 MDa displaying both exo- and endo-proteolytic activities. TppII adopts a toroidal shape of 28 Å large and 60 Å long revealed by electron microscopy data [15] and is considered as the eukaryotic counterpart of Tricorn. Finally, the DppA d-aminopeptidase from *Bacillus subtilis* was the only example of d-stereospecific self-compartmentalizing protease described so far [16]. DppA is a homodecameric particule with *D*_5_ symmetry only found in bacteria and archaea. Here, we report the structure of a novel self-compartmentalizing protease, the Pab87 protein from the hyperthermophilic euryarchaeon *Pyrococcus abyssi*. Pab87 is a serine protease belonging to the MEROPS S12 family and displays sequence similarity with penicillin-recognizing proteins, termed PRP (Figure 1). This proteolytic complex of 0.4 MDa adopts a *D*_4_ barrel-shaped oligomeric architecture with a central channel and an internal cavity. Furthermore, protein Pab87 is, like DppA, d-stereospecific as shown by activity assays and is proposed to play a role in the processing of environmental d-amino acid containing peptides.

## Results and Discussion

**Figure 2:**
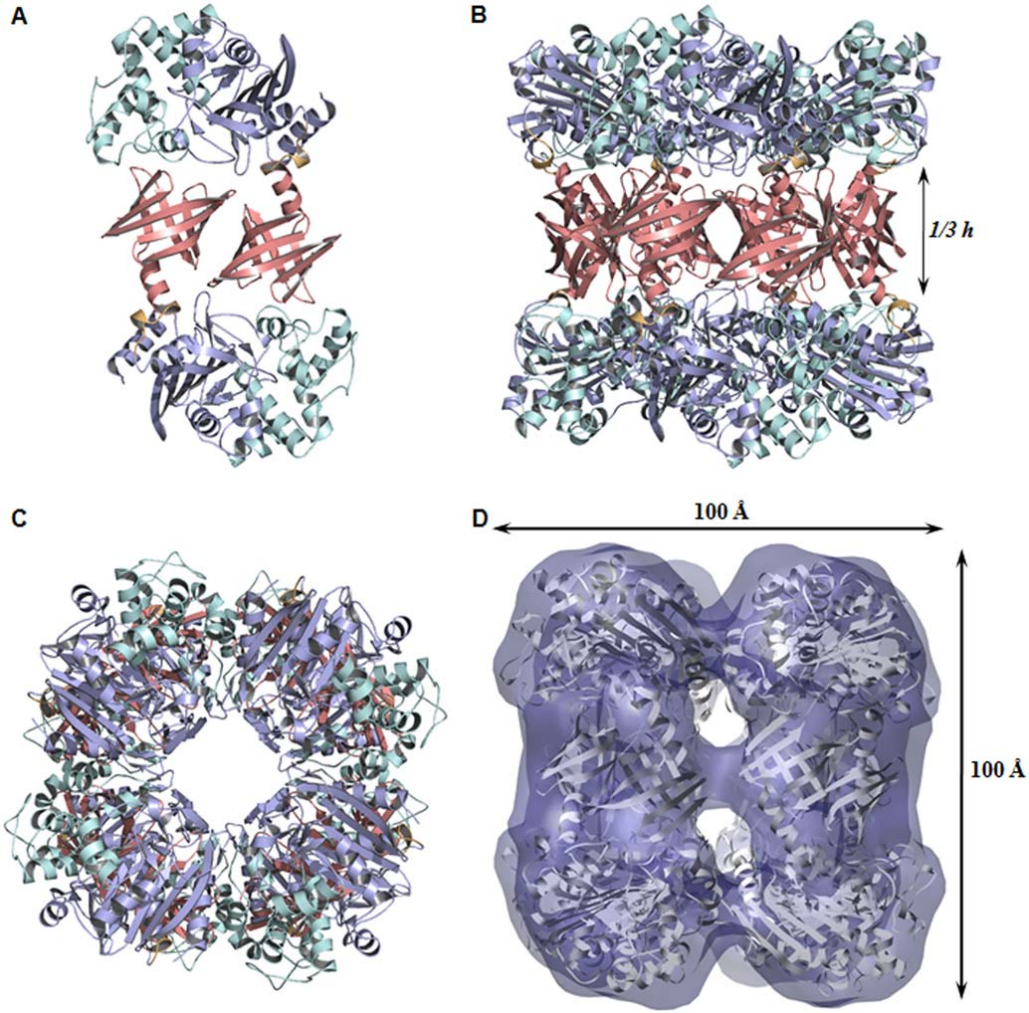
The CubicO particle. Structural regions are color coded as in Figure 1. A, Ribbon representation of the head-to-tail dimer structure forming the edges of the cubic-shaped octamer. B, side view along and C, top view perpendicular to the four-fold axis of the octamer. The central ring formed by the C-terminal domains represents one third of the total height. D, Fitting of cryo-EM map shown in transparent solid surface with the X-ray structure.

The Pab87 structure was determined at 2.2 Å resolution using the single-wavelength anomalous dispersion method with anomalous signal of a high-phasing-power lanthanide derivative, the Lu-HPDO3A complex (Table 1) [17]. Pab87 is the first characterized member of a new self-compartmentalizing protease family, referred to as CubicO proteases as they display a cubic-shaped octameric form (Figure 2). The molecular mass of the macromolecular assembly was confirmed using analytical ultracentrifugation (391±10 kDa). The crystal structure of the *P. abyssi* CubicO protease (Pab87) reveals a 422 symmetry, where the eight monomers are arranged in a barrel-shaped architecture. In the protease complex, monomers associate through a non-crystallographic four-fold axis to form two stacked tetramers that are themselves related by a non-crystallographic two-fold axis perpendicular to the latter. The C-terminal domains interdigitate and form a central ring of one third of the total height of the octamer, playing a central role in this oligomerization (Figure 2B). Moreover, cryo-electron microscopy data show that Pab87 adopts the same octameric structure in solution (Figure 2D).

**Table 1:** Data collection and refinement statistics.

	Lu-HPDO3A derivative
**Data collection**	
X-ray source	ESRF ID29
Wavelength (Å)	1.3403
Temperature (K)	100
Space group	*P*1
Unit-cell parameters (Å, °)	a = 100.94, b = 105.63, c = 112.92, α = 72.17, β = 66.51, γ = 81.39
Resolution range (Å)^a^	30.0 - 2.2 (2.32 - 2.20)
No. of unique reflections	198,616
Multiplicity^a^	7.7 (7.8)
Completeness (%)^a^	95.7 (93.8)
*R*_sym_ a ^, ^ b	0.080 (0.289)
<I/σ(I)> a	17.4 (7.2)
**Refinement**	
Resolution range (Å)	30.0-2.2
*R*_work_/*R*_free_ c	19.6/24.1
Number of atoms	30,885
Protein	28,405
Lu-HPDO3A	112
Water	2,368
Average *B* factor	24.4
R.m.s^d^ deviations from ideal values	
Bond lengths (Å)	0.007
Bond angles (°)	1.3

a Values in parentheses correspond to the highest-resolution shell.

b R_sym_ = Σ**_h_** Σ*_i_* |I**_h_**_,*i*_−<I**_h_**>|/Σ**_h_** Σ*_i_* <I**_h_**>, where I**_h_**_,*i*_ is the *i*th observation of reflection **h** and <*I* **_h_**> is the weighted average intensity for all observations *i* of reflection **h**.

c R_cryst_ = Σ|F_o_−F_c_|/Σ|F_o_|, where F_o_ and F_c_ are observed and calculated amplitudes, respectively. R_free_ is calculated similarly using a test set of reflections. The test set (5% of reflections) is omitted in the refinement.

d Root mean square.

### The CubicO protease family

CubicO protease sequences, found in six deep-sea archaea and one marine γ-proteobacterium, are constituted by two sequence domains, an N-terminal serine protease domain and a C-terminal domain without any detectable sequence similarity (Figure 1). The protease domain displays sequence similarity to PRP, notably the conserved catalytic motifs -SxxK-, -YxN- and -HxG-, and belongs to S12 protease family. The CubicO sequences can be characterized by three additionnal specific motifs, -YAEAF/L- and -FFYLN- located in the N-terminal domain, the latter extending the second PRP catalytic motif, and finally -ERY- found at the extreme C-terminus (Figure 1 and Figure S1).

With regard to the known energy-independent proteases, all CubicO sequences co-exist with at least one TET sequence. In the *P. abyssi* genome, four TET paralogs have been found. While TET aminopeptidases are present in organisms where Tricorn is not found, Tricorn is generally absent within the organisms that possess CubicO proteases. Concerning the d-aminopeptidase DppA, four of these seven organisms possess a DppA sequence. These observations highlight an intricate and complex distribution of either l or d-stereospecific self-compartmentalizing proteases among living organisms [11].

### Pab87 exopeptidase activities

**Figure 3:**
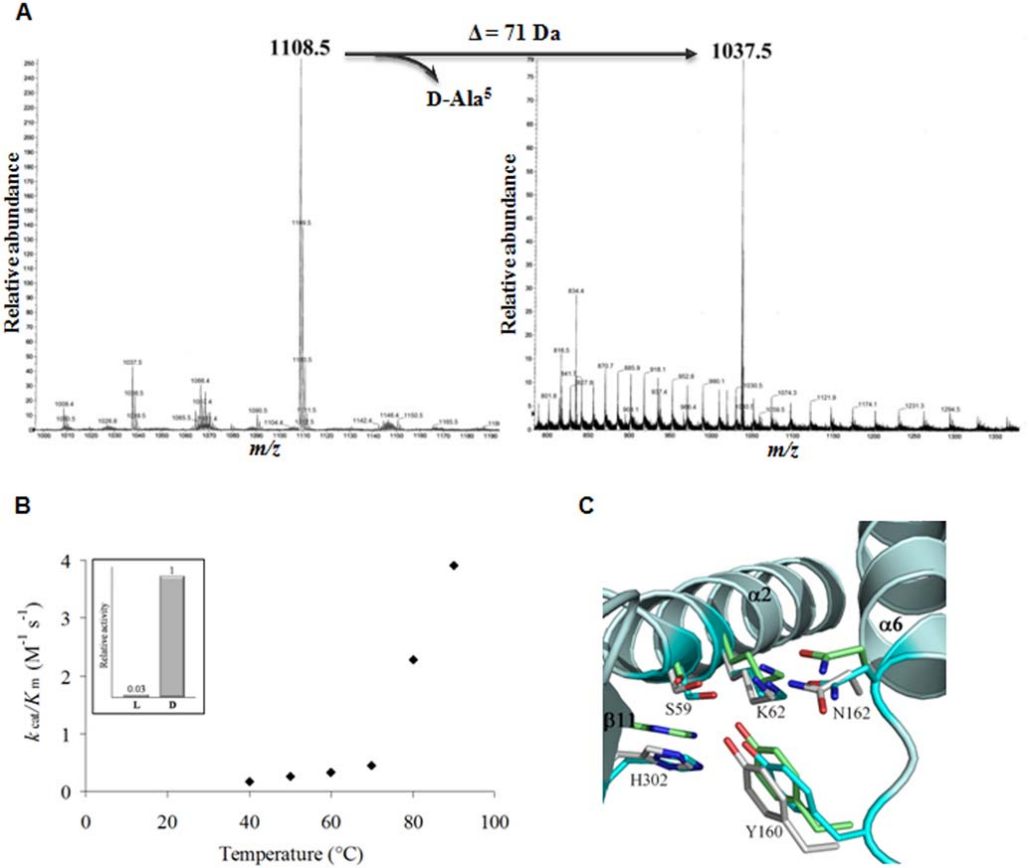
Exopeptidase activities of the CubicO proteases. A, MS analysis of the products of the carboxypeptidation reaction catalyzed by Pab87. Peaks at m/z 1108.5 and 1037.5 correspond to the GM-penta-(Ala-Ala) substrate and to the GM-tetra-(Ala-Ala) product respectively. B, Effect of temperature on the Pab87 catalytic efficiency on d-Ala-*p*-nitroanilide. Box indicates the relative aminopeptidase activity at 90°C of Pab87 on l- and d-*p*-NA. C, Close-up view of the superimposed active sites of Pab87 (in cyan), *O. anthropi* DAP d-aminopeptidase (in white; PDB id 1EI5) and of *Streptomyces* R61 dd-carboxypeptidase (in green; PDB id 3PTE). Only side chains of catalytic residues of the three structures with backbone of Pab87 are represented. Secondary structure elements are indicated in bold.

As Pab87 is a S12 protease, the amino and carboxypeptidase activities were investigated. The aminopeptidase activity was tested using l- and d-Ala-*p*-nitroanilide compounds as substrates. Preliminary studies underlined the high *K*_m_ values for these substrates. Catalytic efficiency *k*_cat_/*K*_m_ could be determined as a function of temperature (Figure 3B). Clearly, the enzyme shows a preference for the substrate with a d configuration. At 90°C, the *k*_cat_/*K*_m_ is 3.92 M^−1^ s^−1^ and 0.06 M^−1^ s^−1^ for respectively the d- and l-Ala-*p*-nitroanilide. The *k*_cat_/*K*_m_ values increase drastically after 70°C. The maximum of activity was obtained at 90°C, the maximum technically reachable temperature, underlying the hyperthermophilic properties of the enzyme. Moreover, dd-carboxypeptidase activity was observed on various muropeptides. Mass spectrometry analyzes clearly show that Pab87 is able to hydrolyse the C-terminal d-alanine of pentapeptidic precursors with or without the sugar moiety from both Gram-negative and Gram-positive bacteria, at 37 and 90°C (Figure 3A). The d-stereospecificity observed for Pab87 is consistent with the activities of the majority of S12 proteases. Catalytic residues of *P. abyssi* CubicO protease perfectly superimpose to those of other S12 members, indicating similar catalytic pathways despite different subtrate specificities (Figure 3C). In conclusion, Pab87 is a hyperthermophilic peptidase that acts on d-amino acid containing peptides at N- and C-termini.

### A novel association of two structural domains

**Figure 4:**
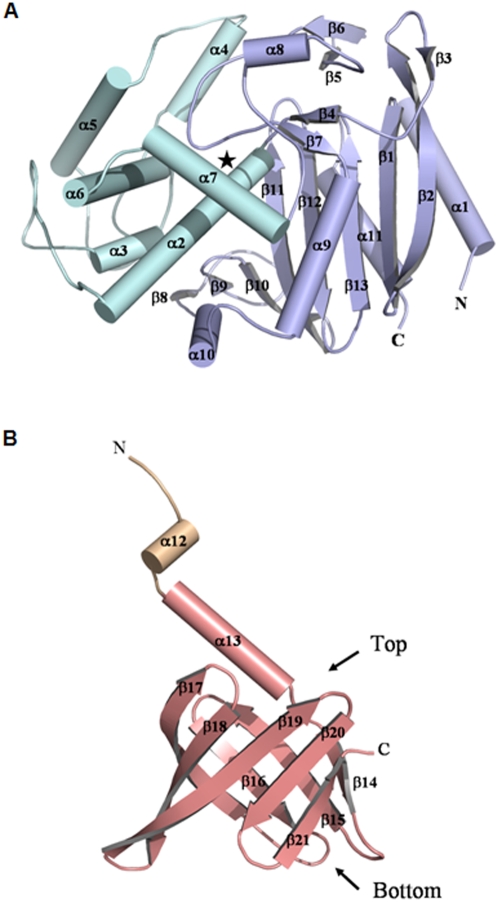
Secondary structure assignation for A, the N- and B, the C-terminal domains of the *P. abyssi* Pab87 monomer, color coded according to Figure 1 (the PRP α/β and all-helical regions are in light blue and cyan, respectively, the linking helix in light orange and the lipocalin domain in salmon). The active site is located by a star in (A).

The 50.4 kDa monomer of Pab87 consists of two structural domains, an N-terminal PRP domain associated to a C-terminal lipocalin domain that plays a crucial role in the octamerization and in the active site compartmentalization (Figure 2A). The N-terminal domain structure follows the pattern of the known PRP structures, composed of two regions, one α/β and one all-helical region with six helices. The α/β region is discontinuous in sequence and consists of a main eight-stranded antiparallel β-sheet flanked by three helices. The C-terminal domain is connected to the N-terminal domain by a small helix, folded back onto the last helix of the N-terminal domain. The Pab87 C-terminal domain presents a typical lipocalin superfold [18] that consists of an α-helix and an eight-stranded antiparallel β-barrel (Figure 4). This N-terminal α-helix closes off the top of the barrel, in vicinity of the PRP domain. Like for all lipocalins, the interior of the barrel is coated with hydrophobic residues. The tunnel observed in the lipocalins is replaced by a small recess at the bottom of the calyx. As lipocalins were first discovered in eukaryotes, and more recently in bacteria [19], the Pab87 C-terminal domain provides the first example of an archaeal lipocalin.

### Structure of the macromolecular assembly

**Figure 5:**
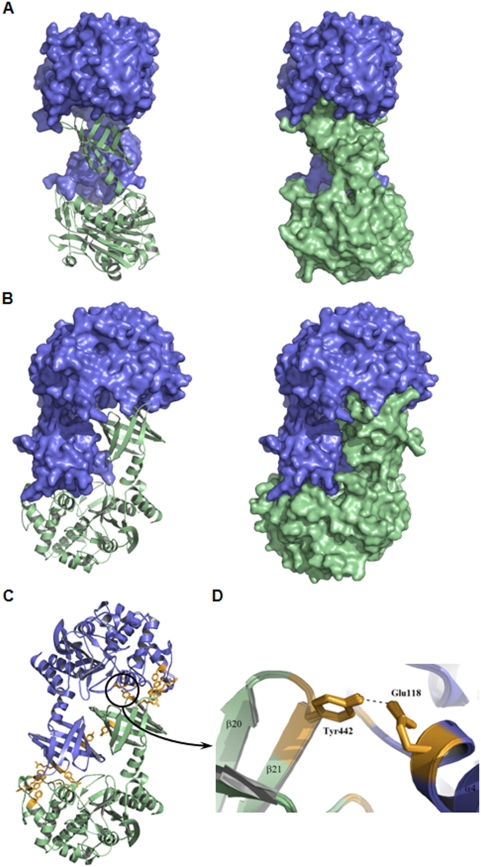
Head-to-tail dimer key-lock interaction. A, Side view of the head-to-tail dimer with the first monomer represented in blue surface and the second in green cartoon (left) and with both monomers represented in surface (right) highlighting the interdigitating of the monomers. B, Same figure as in (A) with a view rotated by 90° to the right. C, Residues involved in short distance interactions between the two monomers are highlighted in orange. D, Hydrogen bond between the two CubicO specific motifs -YAE^118^AF/L- and -ERY^442^-.

The 400 kDa octamer looks like a 100 Å side cube with a central hole along the four-fold axis and small entrances distributed around the equatorial plane of the octamer. The octamer can be considered as four head-to-tail dimers organized around the four-fold axis, forming the edges of the cube (Figure 2). Each monomer interacts with four other subunits of the octamer but main interactions are observed within the head-to-tail dimers, where monomers interact primarily with each other by shape recognition through key-lock type interaction (Figure 5A, B). Intersubunit contacts are formed by electrostatic and hydrogen-bonding interactions between the bottom of the lipocalin domain of the first monomer and the all-helical region of the PRP domain of the second monomer. Three bottom loops of the calyx embrace two helices of the N-terminal domain at the inside and outside face of the octamer. Interestingly, one of these helix-loop interactions links spatially the first and the third CubicO conserved motifs through hydrogen bonding (Figure 5C, D).

Whereas the twelve subunits of the archaeal TET aminopeptidases are organized as a tetrahedron [12], [13], the drosophila TppII protease adopts a toroidal shape [15], the six subunits of the Tricorn protease from *T. acidophilum* associate into rings with *D*_3_ symmetry [9], and the ten monomers of DppA d-aminopeptidase from *B. subtilis* oligomerize in a *D*_5_ symmetry [16], the structure of the *P. abyssi* CubicO protease reveals a novel giant protease architecture, a *D*_4_ barrel-shaped oligomer with a central channel and an internal cavity.

### The proteolytic central chamber

**Figure 6:**
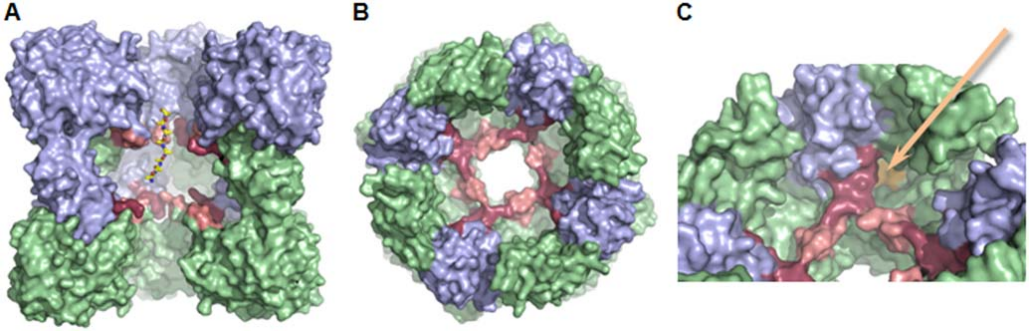
The proteolytic chamber. A, Open side view of the octamer. The front head-to-tail dimer has been removed. The top and bottom tetrameric rings are colored in blue and green, respectively. The β5–β6 and β16–β17 loops forming the two crown-shaped surfaces are colored in salmon and raspberry, respectively. To illustrate the size of the cavity, an Ala_8_-peptide was manually placed in extended conformation. B, Open top view of the octamer down the four-fold axis. For better clarity, the four N-terminal PRP domains of the top tetramer and the four C-terminal domain β16–β17 loops of the bottom tetramer have been removed. C, Same figure as in (B) tilted so that the active site entrance is visible. Catalytic pocket is highlighted in orange.

**Figure 7:**
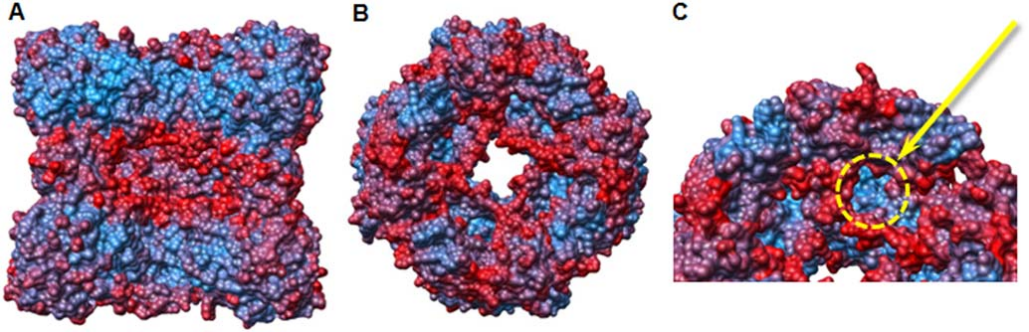
Relative flexibility of the octamer. The structure is colored by B-factors to illustrate flexibility (dynamic disorder). Red color reflects high flexibility, light blue reflects low flexibility relative to the B-factors of the overall complex. A, Open side view of the octamer. The front head-to-tail dimer has been removed. The β5–β6 and β16–β17 loops forming the two crown-shaped surfaces appear highly flexible. B, Open top view of the octamer down the four-fold axis. For better clarity, the four N-terminal PRP domains of the top tetramer and the four C-terminal domain β16–β17 loops of the bottom tetramer have been removed. C, Same figure as in (B) tilted so that the active site entrance is visible. Catalytic pocket is highlighted in yellow.

The 20 Å wide square-shaped channel, running along the four-fold axis through the entire complex, joins the equatorial two-fold axes into a 60 Å wide central cavern formed by association of the eight C-terminal domains (Figure 6A). Each active site, facing the central cavern, lies between three domains, the PRP and the lipocalin domains of one monomer, and the second lipocalin domain of a head-to-tail dimer (Figure 6C). The inside volume of the cavity is about 80,000 Å^3^, large enough to accomodate small unfolded peptides (Figure 6), and reminds those of other self-compartmentalizing proteases known to bury their active sites in an inner cavity. In the *P. abyssi* CubicO protease, the proteolytic chamber is separated from the access channels by two crown-shaped surfaces at about 10 Å of each side of the equatorial plane. These crowns are formed by two loops of opposite monomers joining at the same height (Figure 6A). The shape and size of the cavity are conserved in all CubicO proteases as shown by the structure modelling of the six other members of the family (Figure S2). Variability mainly concerns the access to the active site through the large and flexible loops forming the inside crowns appearing to function as substrate sieves (Figure 7).

### Conclusion

The presence of such proteases with d-stereospecificity in archaea raises the question of their physiological function as these organisms generally lack d-amino acid containing peptides. All organisms that possess CubicO proteases are found near deep-sea hydrothermal vents. In these biotopes, archaea and bacteria live in intimate and complex symbiosis [20]. Growing bacteria continuously release muropeptide fragments of their cell wall into the extracellular milieu that could serve as nutrients for other organisms living in the same habitat. As it has been shown for *Pyrococcus* species, heterotrophic organisms are able to utilize amino acids as sole carbon and nitrogen sources [21]. Analyses of the genomic context of the Pab87 gene revealed that it is surrounded by genes involved in the amino acid/peptide transport or metabolism, e.g. a PLP-dependent aminotransferase (PAB0086), a d-aminoacylase (PAB0090) and the dipeptide ABC transporter (PAB0091-95). Taken all together, we propose that CubicO proteases play a central role in proteolysis of these bacterial cell debris that contains d-amino acids.

## Materials and Methods

### Multiple sequence alignment

Pab87 CubicO homologs were detected using iterative searches of the non-redundant database of protein sequences at the NCBI with the PSI-BLAST program and the C-terminal domain of Pab87 as the query. The initial multiple alignment of complete sequences was performed using PipeAlign [22] and manually adjusted to maximize the sequence conservation.

### Protein production and purification

Production and purification of Pab87 were carried out as previously described [23]. Briefly, Pab87 was purified by heating at 90°C, and using anion exhange and size exclusion chromatographies. The protein was concentrated in 50 mM Tris-HCl buffer pH 8.5, 350 mM NaCl.

### Analytical ultracentrifugation

Sedimentation velocity experiments were performed in a Beckman XL-A analytical ultracentrifuge using a double sector charcoal-Epon cell at 20°C and 40,000 rpm. Absorbance scans were taken at 280 nm every 1 min. The protein concentration was 10 µM in 150 mM Tris (pH 8.5), 350 mM NaCl. The program Sednterp [24] was used to calculate solvent density (1.01689 g/cm), solvent viscosity (0.01083 Poise) and partial specific volume (0.7499 ml/g) using the amino-acid composition. The sedimentation data were analyzed with the program Sedfit [25] using the continuous c(s) and c(M) distributions, and showed that 94% of the protein sedimented with a corrected sedimentation coefficient s_20°C,w_ = 14.71±0.22 S. The estimated frictional ratio (f/f_0_ = 1.25) is consistent with a globular shape of the particle. The molecular mass of the particle (391,420±10,670 Da) is in very good agreement with the calculated molecular mass of the octamer (403,542 Da).

### Cryo-electron microscopy

Pab87 was diluted to 0.5 mg mL^−1^ in 50 mM Tris-HCl buffer pH 8.5, 350 mM NaCl. 5 µL of this solution was applied onto an electron microscopy grid covered with a holey carbon film. The grid was plunged into an ethane slush cooled with liquid nitrogen. The frozen hydrated specimen were observed at low temperature on a cryo electron microscope (Tecnai F20, FEG cathode operated at 200 kV) and images were recorded under low-dose condition (less than 2,000 electrons/nm^2^) on Kodak SO163 photographic plates. Areas covered with individual molecules were recorded at a 50,000 times magnification. The micrographs were digitized with a 5 µm raster size using a drum scanner (Primescan D7100, Heidelberg). The data was coarsened twice resulting in a final pixel spacing of 0.254 nm. Boxing and CTF phase correction of the 6,480 molecular images were performed in the EMAN software package. Image analysis was performed using the IMAGIC software package [26] (Image Science Software, Berlin, Germany) as described earlier [27]. The resolution of the final reconstructions was estimated to be around 14 Å from the Fourier shell correlation function obtained by comparing two reconstructions, generated by splitting randomly the data set in half and according to the 0.5 cut-off in the Fourier shell correlation curve (0.5 FSC criterion [28]). Figure 2D and Figure 7 were prepared with the UCSF Chimera package from the Resource for Biocomputing, Visualization, and Informatics at the University of California, San Francisco (supported by NIH P41 RR-01081) [29] (http://www.cgl.ucsf.edu/chimera).

### Crystallization and diffraction data collection

Pab87 (7 mg mL^−1^) was mixed with lanthanide complex solution at required concentration to obtain Lu-HPDO3A final concentrations of 50, 100 and 200 mM. Pab87/Lu-HPDO3A complex was co-crystallized by hanging drop vapor diffusion method with 20 mM CaCl_2_, 30% MPD and 100 mM sodium acetate buffer pH 4.6 at 18°C. Diffraction data from a crystal obtained with Lu-HPDO3A concentration of 200 mM was used for SAD data collection at 2.2 Å resolution on ID29 beamline (ESRF, Grenoble, France). The wavelength was set to 1.3403 Å corresponding to the lutetium *L*_III_ absorption edge (*f*″∼28 e^−^). Data were processed with XDS [30] and scaled with SCALA from the *CCP4* suite [31]. The crystal has the symmetry of the space group *P*1 with eight molecules per asymmetric unit (see Table 1 for crystallographic parameters).

### Structure determination and refinement

AutoSHARP [32] was used to locate the lutetium sites based on the Lu-derivative anomalous data and to generate initial SAD phases at 2.2 Å resolution with a figure of merit of 0.35. Phases further improved by density modification, with a figure of merit of 0.88, yielded a clearly interpretable map that was submitted to automatic building using BUCCANEER [33]. The resulting built model corresponding to 89% of the structure was completed using COOT [34] and refined with CNS [35]. The R-factor of the final model is 0.196 (R_free_ = 0.241), using all data from 30.0 to 2.2 Å resolution (Table 1). The model contains amino acid residues 1–447 for chains A, B, F, G, H, 1–446 for chains C, D, E, 4 HPDO3A, 17 lutetium ions and 2,368 water molecules. The average root mean square deviation of the NCS-related subunits is 0.37 Å for the 446 superimposed Cα atoms. Model quality and stereochemistry were checked using CNS and PROCHECK [36] (Table 1). Atomic coordinates and structure factors of protein Pab87 in complex with Lu-HPDO3A have been deposited in the Protein Data Bank with accession code 2QMI.

### Molecular modelling

The monomeric structure of the CubicO proteases from *Pyrococcus horikoshii*, *Caldivirga maquilingensis*, *Hyperthermus butylicus*, *Pyrobaculum aerophilum*, *Aeropyrum pernix* and marine γ-proteobacterium HTCC2207 were modelled by SWISS-MODEL [37] using the 3D structure of one Pab87 monomer from *Pyrococcus abyssi* as model (PDB id 2QMI). The octamers were generated using the same *D*_4_ symmetry than the Pab87 octamer and optimized through rigid body minimization. Minimization process was pursued using simulated annealing and energy minimization with CNS [35]. The geometric quality of the models was assessed with Procheck [38]. The models were considered to be good with Verify3D [39], ProsaII [40] and Eval23D [41].

### Activity assays

The d- and l-aminopeptidase activities were detected by monitoring the formation of *p*-nitroaniline from d- or l-Ala-*p*-nitroanilide at 405 nm in a 20 mM Tris buffer (pH 8.0) from 40 to 90°C by 10°C steps [42].

The d-Ala-d-Ala carboxypeptidase activity was assayed on GlcNAc-MurNAc-l-Ala-γ-d-Glu-l-(*N* ^ε^-d-Asp)Lys-d-Ala-d-Ala, GlcNAc-MurNAc-l-Ala-γ-d-Glu-l-(*N* ^ε^-l-Ala-l-Ala)Lys -d-Ala-d-Ala, GlcNAc-MurNAc-l-Ala-γ-d-Glu-*meso*DAP-d-Ala-d-Ala and l-Ala-γ-d-Glu-l-Lys-d-Ala-d-Ala. The assays were performed in a 10 µL mixture containing protein Pab87 (5 µg), Tris-HCl (20 mM, pH 8.0) and substrate (1 mM). The reaction mixtures were incubated for 90 min at 37 or 90°C, and desalted using a micro column (ZipTipC_18_; Millipore). The products of the reaction were analyzed by nanoelectrospray MS in the positive mode (Qstar Pulsar I; Applied Biosystems). The disaccharide-pentapeptide substituted by a d-aspartate residue (l-Lys^3^-d-Asp) was purified from the peptidoglycan of *Enterococcus faecium* M512 [43]. The disaccharide-pentapeptide substituted by an l-Ala-l-Ala side chain (l-Lys^3^-l-Ala-l-Ala) was purified from *Enterococcus faecalis* JH2-2 [44] and the disaccharide-pentapeptide containing *meso*-diaminopimelic acid (*meso*DAP^3^) was purified from *E. coli* strain ATCC 25113, a strain deleted for several dd-carboxypeptidases. The procedures used for peptidoglycan preparation, digestion with muramidases, and reduction of MurNAc to muramitol with sodium borohydride have been previously described for enterococci [43] and *E. coli* [45]. The resulting muropeptides, disaccharide-tetrapeptides or tetrapeptide formed by the hydrolysis of the C-terminal d-Ala residue, were separated by RP-HPLC in acetonitrile gradients containing trifluoroacetic acid [43] and identified by mass spectrometry (MS). The concentration of the muropeptides was estimated by amino acid analysis after acidic hydrolysis with a Hitachi autoanalyzer [46]. Pentapeptide l-Ala-γ-d-Glu-l-Lys-d-Ala-d-Ala was purchased from Sigma.

## Supporting Information

Figure S1 Structure-Corrected Sequence Alignment of CubicO protease family members. The sequence names are as following: pab87, *Pyrococcus abyssi* PAB0087, phori, *Pyrococcus horikoshii* PH0142/PH0143, cmaqu, *Caldivirga maquilingensis* Cmaq_0116, paero, *Pyrobaculum aerophilum* PAE3237, hbuty, *Hyperthermus butylicus* Hbut_1035, apern, *Aeropyrum pernix* Ap_0338, gprot, marine γ-proteobacterium HTCC2207. α-helices (cylinders) and β-strands (arrows) of *P. abyssi* Pab87 are aligned with the sequences and color coded according to Fig 1A (the peptidase α/β and all-helical regions are in light blue and cyan, respectively, the linking helix in light orange and the lipocalin domain in salmon). Residues emphasized by black shading are 100% conserved, and gray shading represents 85% conservation in the multiple alignment of the 7 sequences. CubicO specific motifs are underlined by red stars (the YAEAF/L, FFYLN and ERY are located at residues 116–120, 158–162 and 440–442, respectively, in Pab87) and PRP specific motifs by green stars (the SXXK, YXN and HXG motifs are located at residues 59–62, 160–162 and 302–304, respectively, in Pab87).
(0.09 MB PDF)

Figure S2 Structure of the CubicO protease models from A, *Aeropyrum pernix*, B, *Caldivirga maquilingensis*, C, the marine γ-proteobacterium HTCC2207, D, *Hypethermus butylicus*, E, *Pyrobaculum aerophilum*, F, *Pyrococcus abyssi*, G, *Pyrococcus horikoshii*. On left, open side view of the octamers. To visualize the internal cavity, the head-to-tail dimer in the front of the picture has been removed. The top and bottom tetrameric rings are colored in blue and green, respectively. The beta 5-beta 6 and beta 16-beta 17 loops forming the two crown-shaped surfaces are colored in salmon and raspberry, respectively. On right, ribbon representation of the octamers.
(1.85 MB PDF)
