# Supplementary material for: Structure of the Archaeal Pab87 Peptidase Reveals a Novel Self-Compartmentalizing Protease Family
Source: PLoS One. 2009 Mar 5;4(3):e4712. doi: 10.1371/journal.pone.0004712 (PMC2651629; doi:10.1371/journal.pone.0004712)
Supplement: Figure S1 — Structure-Corrected Sequence Alignment of CubicO protease family members. The sequence names are as following: pab87, Pyrococcus abyssi PAB0087, phori, Pyrococcus horikoshii PH0142/PH0143, cmaqu, Caldivirga maquilingensis Cmaq_0116, paero, Pyrobaculum aerophilum PAE3237, hbuty, Hyperthermus butylicus Hbut_1035, apern, Aeropyrum pernix Ap_0338, gprot, marine γ-proteobacterium HTCC2207. α-helices (cylinders) and β-strands (arrows) of P. abyssi Pab87 are aligned with the sequences and color coded according to Fig 1A (the peptidase α/β and all-helical regions are in light blue and cyan, respectively, the linking helix in light orange and the lipocalin domain in salmon). Residues emphasized by black shading are 100% conserved, and gray shading represents 85% conservation in the multiple alignment of the 7 sequences. CubicO specific motifs are underlined by red stars (the YAEAF/L, FFYLN and ERY are located at residues 116–120, 158–162 and 440–442, respectively, in Pab87) and PRP specific motifs by green stars (the SXXK, YXN and HXG motifs are located at residues 59–62, 160–162 and 302–304, respectively, in Pab87). (0.09 MB PDF) [file pone.0004712.s001.pdf]

Figure S1. Structure-Corrected Sequence Alignment of CubicO protease family members. The sequence names are as following: **pab87**, *Pyrococcus abyssi* PAB0087, **phori**, *Pyrococcus horikoshii* PH0142/PH0143, **cmaqu**, *Caldivirga maquilingensis* Cmaq_0116, **paero**, *Pyrobaculum aerophilum* PAE3237, **hbuty**, *Hyperthermus butylicus* Hbut_1035, **apern**, *Aeropyrum pernix* Ap_0338, **gprot**, marine γ-proteobacterium HTCC2207. α-helices (cylinders) and β-strands (arrows) of *P. abyssi* Pab87 are aligned with the sequences and color coded according to Fig 1A (the peptidase α/β and all-helical regions are in light blue and cyan, respectively, the linking helix in light orange and the lipocalin domain in salmon). Residues emphasized by black shading are 100 % conserved, and gray shading represents 85 % conservation in the multiple alignment of the 7 sequences. CubicO specific motifs are underlined by red stars (the -YAEAF/L-, -FFYLN- and -ERY- are located at residues 116-120, 158-162 and 440-442, respectively, in Pab87) and PRP specific motifs by green stars (the -SXXK-, -YXN- and -HXG- motifs are located at residues 59-62, 160-162 and 302-304, respectively, in Pab87).

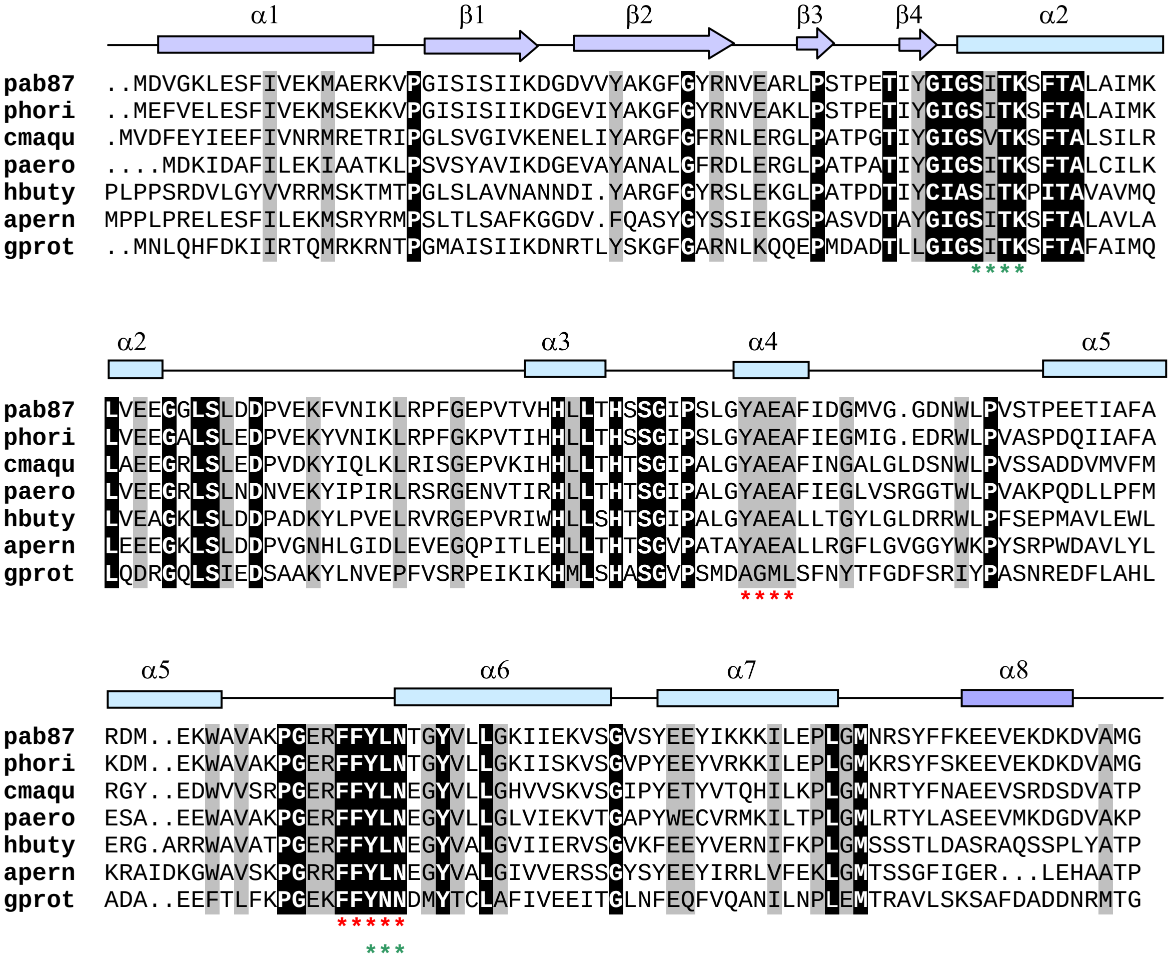

```
       β5         β6                β7  α9                  α10    β8
pab87  YILDKEGR...LVPQPFPYG..ITADGGLLSSVLDLAKYLKMYIER....DESIVSKEYIEKMETSYIKVPWE
phori  YIVDKEGK...LIPQGFPYG..ITSDGGLLSNVLDLAKYLKMYMER....DEKVVSKESIEAMEKPYIKVPWE
cmaqu  YILDRKGR...HIPSRFPFG..ITADGGLLSNVIDMSRYITMLMNRGVLNDVRIVSKDYLELAERRYINVPWR
paero  YYPDPEKGT..PTPGRIPIG..ITSDGGIMSNALDMAKYVAMLINRGTYNGVEIIGKKSVEEAEIKRVAVPWR
hbuty  YDASTTP....PQPAMIPTG..ITADGGAYSTVLDLVKFMSMLANRGRLGDTEILSPSSVEEMEKPRVQLPSQ
apern  YKPG..DR...LEPVPLPAG..ITADGGIMSTARDMLRYVESLARG....EF....RGYAERMEKPRVRVPWQ
gprot  YLLEMKNGKSCAKESDVPIDGYLQAIGGLYVSMNEMLNYAKCLLNHGEFNGTQILSKESVATLFASQIATPYG
```

```
           β9     β10      β11          β12       β13         α11
pab87  IFGGEGYGYGLIIYP..NFLGEKLVGHSGSVGMYTGYIGYIPEKKIGVAVLENSSGYPPSYIAMYALALLLGK
phori  LFGGESYGYGLIIYP..NFLGEKLIGHSGSVGMYTGYIGYIPEKQIGVAVLENSSGYPPSYIGMYALALLLDK
cmaqu  IIGDEGYGYGLIVSE..NFFGRKLVQHSGNVLVYTAYMAYLPSDRLGVVIMSNAEGYSMMKMGIYILTRLVGH
paero  LLGDEGYGYGLVISE..NFYGRKIVMHGGNVLVYTAHMAYLPYDRIGVVVLANSTGYPMSYIAMYILAIALGR
hbuty  IWGNDSYGLGLIIYD..GFPGGRLVGHSGSVYVHTGFAGYIRGKSVIVSVLANADPG.ATTIGMALAADAAGI
apern  ALGGEAYGYGLIIYP..DFMGGRLASHGGSLLAYTAWMGYSTRLDSGVVLLSNTTGYPLAAMGMAVLSVLSGG
gprot  EGNNPQYALGWCIESPTNFVPYGVLQHGGGMGTSNSFLLLIPELNAAVVAAENAGTGITPLIARVAVAELLGD
                                 ***
```

```
       α12   α13          β14      β15      β16           β17
pab87  NPEKELPFIYRERILKKVEGRYMGYKGTIKFEVKVDGDVVYLRALGRAFTYTIPLFPEVLEED........FI
phori  NPEEELPFLSREKKLKRIEGLYKGYKGTLKFEVKVEGDIVYLKFLGKIFTYMVPLFPEVLEED........FI
cmaqu  EPRELWAFKL.EDTLRRLEGVYEAYNGTVRVSVKARGDFLVIKSSTRYTEESTILIPEEVSGD........YA
paero  NPE.ELPFLMREKVLRKLEGTYRGYKGTVTYTAKAKGDILILKSQ...WGEELYLFPEEVRED........YA
hbuty  DYHV.LPMVRAEEAAEKLEGIYHGYEGTVRFRVKALGDALVIESLSR.PGLQEVMVPERIEPP........VY
apern  SHLDPKPVRA.LEIVERLEGYYEGFDSSIAFNIRRVGTGLLVEPVGGVG.SPMALSPREIPGPGELEAGEGSV
gprot  KPEVVVEDLRMDLCLAEVEGTYQSQYDMYKLTVGRKNGVLQADVETDDGSFSFPLIPSDIDNL........EF
```

```
        β18       β19        β20    β21
pab87  KCYTLSNGRKMYAEFYIKDNKVDLIFERYRLIKS.........
phori  KCYTLSNGRKMYAEFYIKDNTVELLFERYKLVKG.........
cmaqu  RFYTLLYGAKVPVEFTIKGGKIYMTYERYVFIKRE........
paero  KFHTYTKGYKLDVEFHIGKDGVKIVFERYLLVKSA........
hbuty  EYTVYRGGRRMKAVFTR..........................
apern  VFVSPYMGGVLEVEFNWSGERVEALLERYRLVKRGPARMPGV.
gprot  SVYSLRSSRHAKVVFYRDEKTQKIAFSRYDRFMYRRV......
                                  ***
```
