# Supplementary material for: Structure of the Archaeal Pab87 Peptidase Reveals a Novel Self-Compartmentalizing Protease Family
Source: PLoS One. 2009 Mar 5;4(3):e4712. doi: 10.1371/journal.pone.0004712 (PMC2651629; doi:10.1371/journal.pone.0004712)

Figure S2. Structure of the CubicO protease models from **A**, *Aeropyrum pernix*, **B**, *Caldivirga maquilingensis*, **C**, the marine γ-proteobacterium HTCC2207, **D**, *Hypethermus butylicus*, **E**, *Pyrobaculum aerophilum*, **F**, *Pyrococcus abyssi*, **G**, *Pyrococcus horikoshii*. On left, open side view of the octamers. To visualize the internal cavity, the head-to-tail dimer in the front of the picture has been removed. The top and bottom tetrameric rings are colored in blue and green, respectively. The β5-β6 and β16-β17 loops forming the two crown-shaped surfaces are colored in salmon and raspberry, respectively. On right, ribbon representation of the octamers.

A *A. pernix*

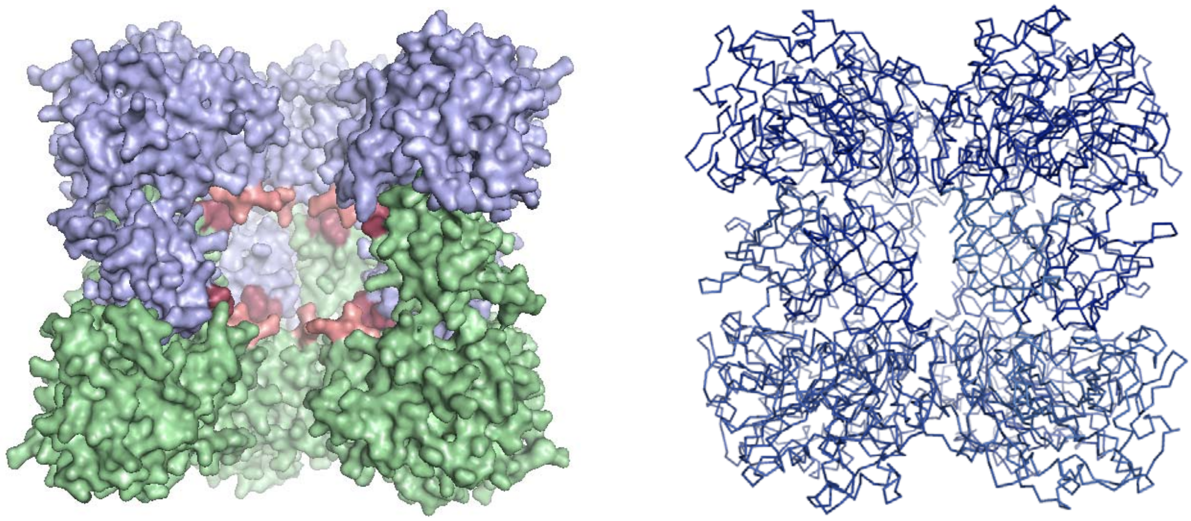

B *C. maquilingensis*

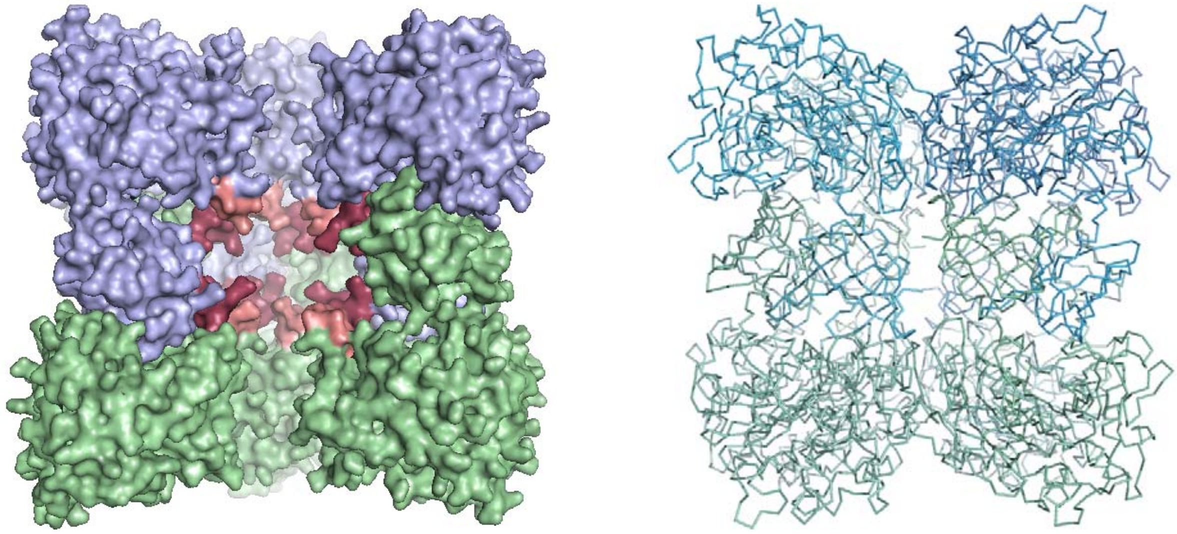

C marine γ-proteobacterium HTCC2207

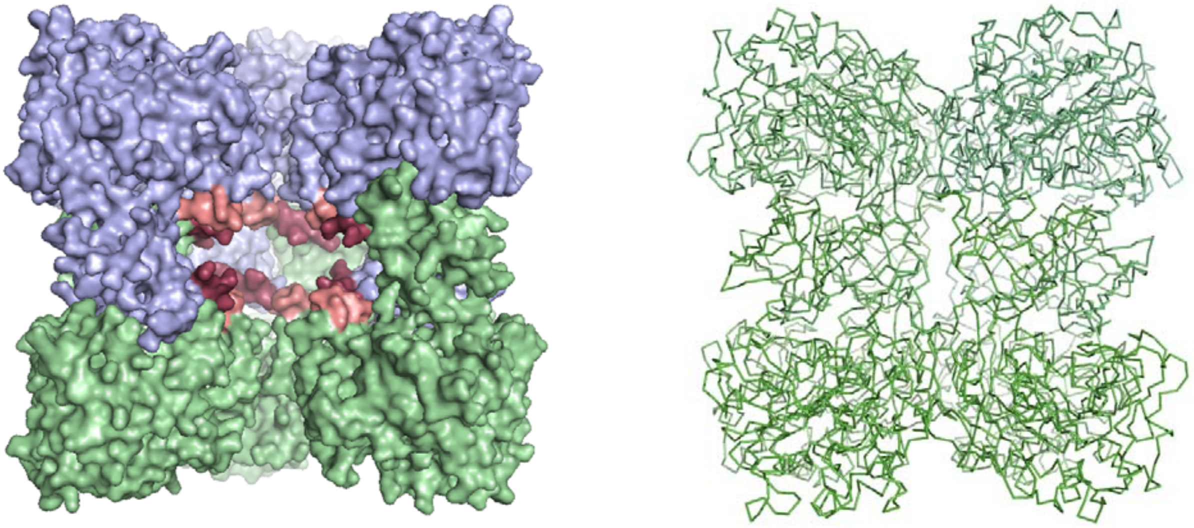

D *H. butylicus*

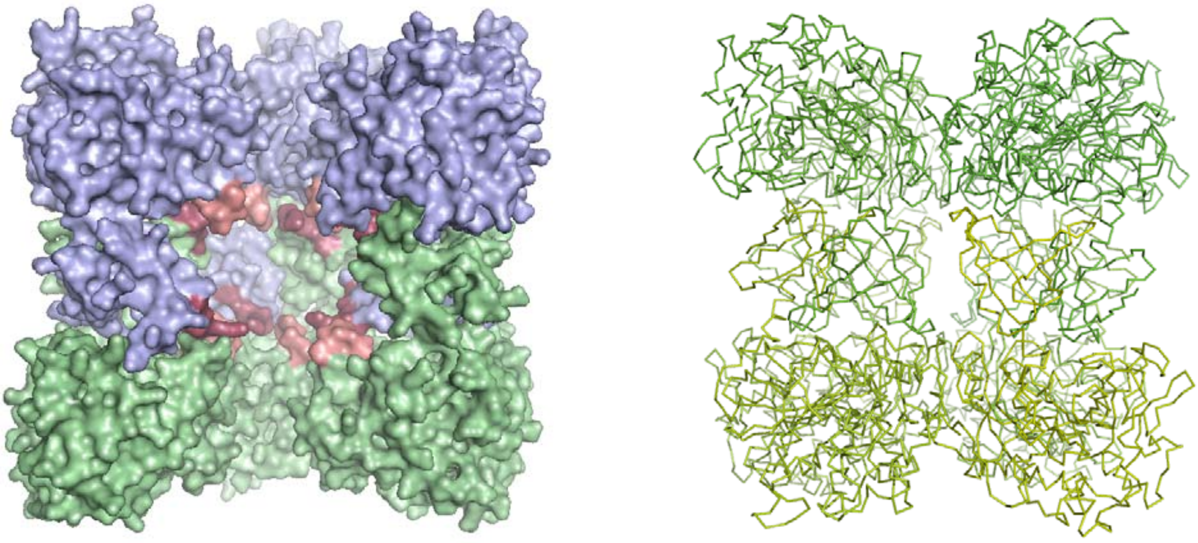

E *P. aerophilum*

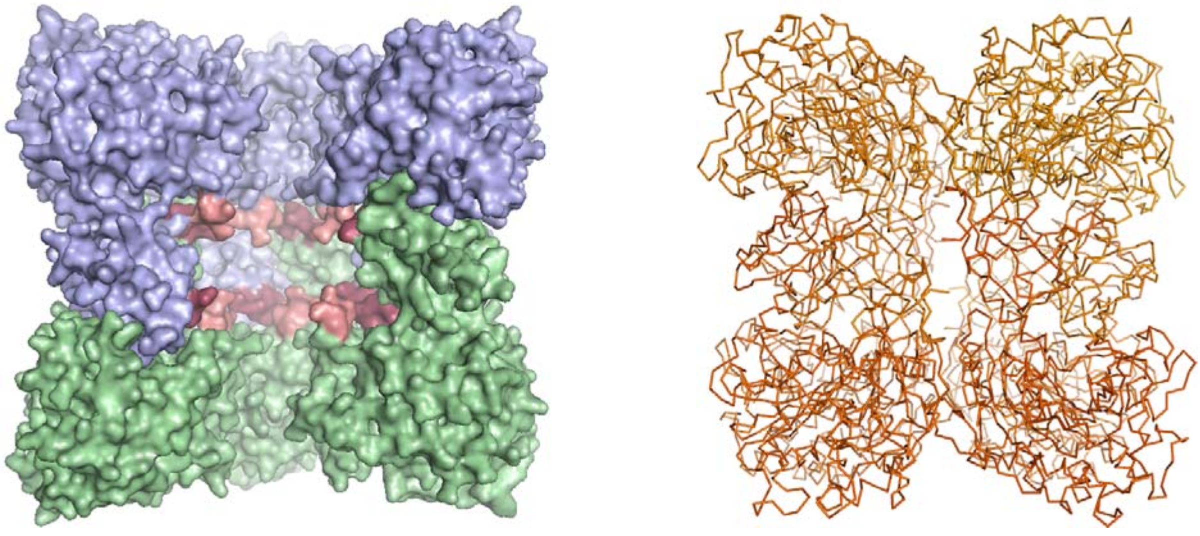

F *P.abyssi*

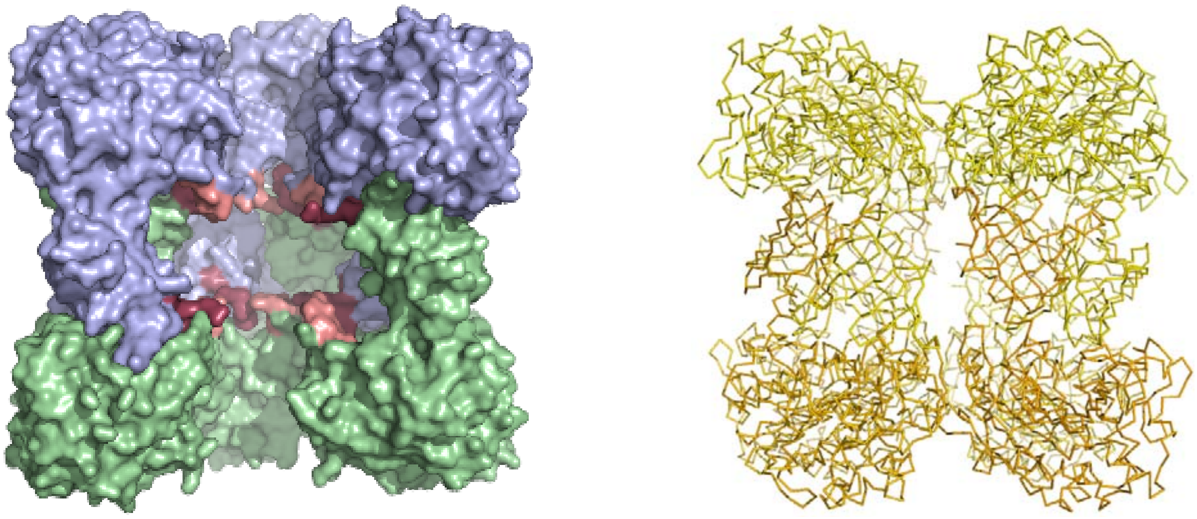

G *P.horikoshii*

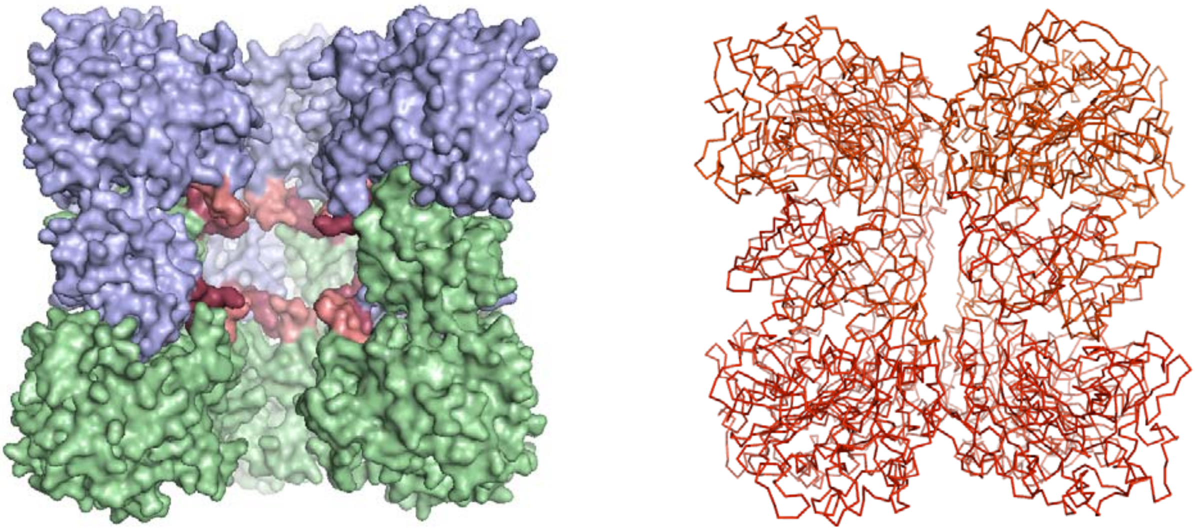

Supplement: Figure S2 — Structure of the CubicO protease models from A, Aeropyrum pernix, B, Caldivirga maquilingensis, C, the marine γ-proteobacterium HTCC2207, D, Hypethermus butylicus, E, Pyrobaculum aerophilum, F, Pyrococcus abyssi, G, Pyrococcus horikoshii. On left, open side view of the octamers. To visualize the internal cavity, the head-to-tail dimer in the front of the picture has been removed. The top and bottom tetrameric rings are colored in blue and green, respectively. The beta 5-beta 6 and beta 16-beta 17 loops forming the two crown-shaped surfaces are colored in salmon and raspberry, respectively. On right, ribbon representation of the octamers. (1.85 MB PDF) [file pone.0004712.s002.pdf]
